# Pallister–Killian syndrome in a two‐year‐old boy

**DOI:** 10.1002/ccr3.892

**Published:** 2017-04-08

**Authors:** Leigh Stone, Ramya Tripuraneni, Michelle Bain, Claudia Hernandez

**Affiliations:** ^1^ Department of Dermatology University of Illinois at Chicago 808 South Wood Street Room 376 CME Chicago 60612 Illinois USA; ^2^ Department of Dermatology Northwestern University 676 North St. Clair Street Suite 1600 Chicago 60611 Illinois USA

**Keywords:** Developmental defects, dyspigmentation, Pallister–Killian syndrome

## Abstract

Pallister–Killian syndrome (PKS) is a rare, sporadic, multisystem developmental disorder characterized by craniofacial dysmorphic features. We report a case of a two‐year‐old boy with PKS to highlight the cutaneous findings and emphasize the importance of diagnostic skin biopsies in patients with cutaneous pigmentation changes and distinctive facial features.

## Graphical abstract

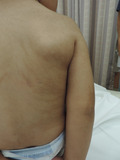

## Introduction

Clinical manifestations of Pallister–Killian syndrome (PKS) include characteristic craniofacial dysmorphism, congenital heart defects, diaphragmatic hernia, hypotonia, epilepsy, and cutaneous pigmentary anomalies. It was first described in adults by Pallister in 1977 and again in 1981 by Killian and Teschler‐Nicola in children with dysmorphic features, mental retardation, and cutaneous findings 1, 2. It is sometimes referred to as Pallister mosaic syndrome, Killian syndrome, Killian/Teschler‐Nicola syndrome, Teschler‐Nicola/Killian syndrome, tetrasomy 12p, and isochromosome 12p syndrome. PKS is the result of extra copies of the short arm of chromosome 12 and demonstrates a tissue‐limited mosaic pattern. Patients with PKS have a population of karyotypically normal cells and another set of cells containing the supernumerary isochromosome 12p. In spite of our improved understanding of the molecular etiology of PKS, the mechanism by which the 12p isochromosome results in congenital anomalies remains unclear.

## Case Description

A two‐year‐old boy was referred to dermatology for evaluation of congenital skin findings that have remained constant since birth. He was born at term after an uncomplicated pregnancy to a 32‐year‐old mother. He was noted soon after birth to have trouble swallowing his milk and suffered recurrent aspiration pneumonias prompting speech and occupational therapy evaluation. The patient was also noted to have “episodes” suspicious of epilepsy and underwent a brain MRI at 7 months which was normal as well as an electroencephalogram (EEG) at 8 months which did not record any epileptiform activity but mild bilateral cerebral dysfunction without specific etiology. Despite the EEG findings, his episodes were highly suspicious of epilepsy and levetiracetam was initiated. He experienced developmental delay in gross motor skills, became easily overstimulated in crowds, had hyperacusis, and displayed atypical behaviors flapping and flicking his fingers. In addition, his neurologist and pediatrician noted plagiocephaly, intermittent alternating exotropia, and “hyperpigmented skin” on his back and extremities. His family history was significant for a maternal aunt and uncle with developmental delay and epilepsy as well as a brother with learning disabilities. His pediatrician performed metabolic screens including plasma amino acids, which showed a mild nondiagnostic elevation of branched‐chain amino acids and normal urine organic acids. He was sent to genetics where his evaluation included microarray of peripheral blood, which was normal. Given the presence of skin findings, he was sent to dermatology for evaluation and biopsy. Genetics suspected PKS given his presentation and negative workup to that point.

**Figure 1:**
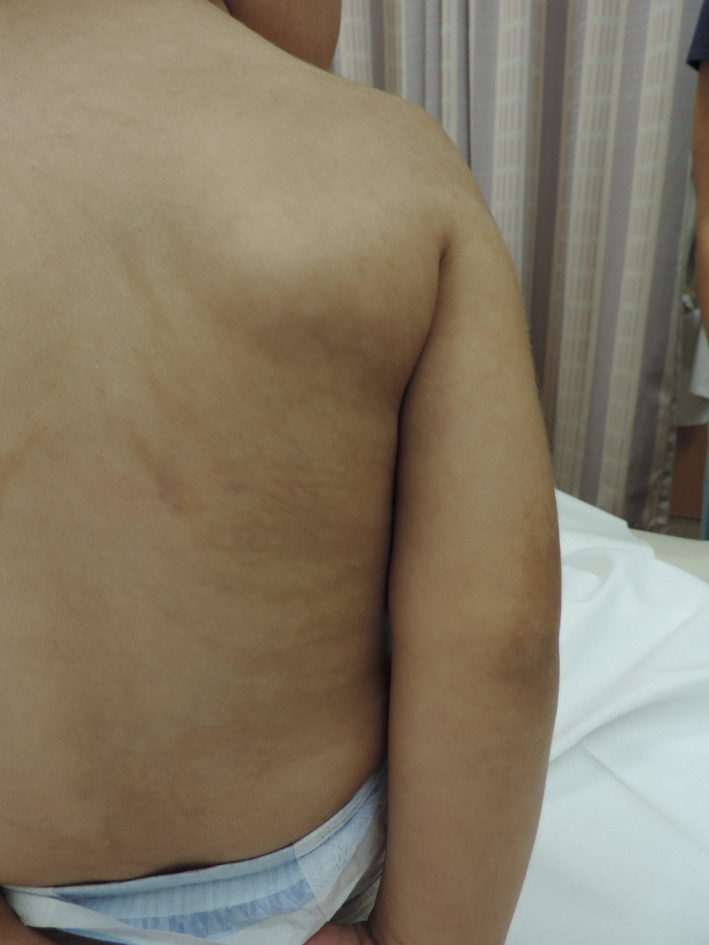
Blaschkoid hypo‐ and hyperpigmented streaks present on the trunk and upper extremity.

**Figure 2:**
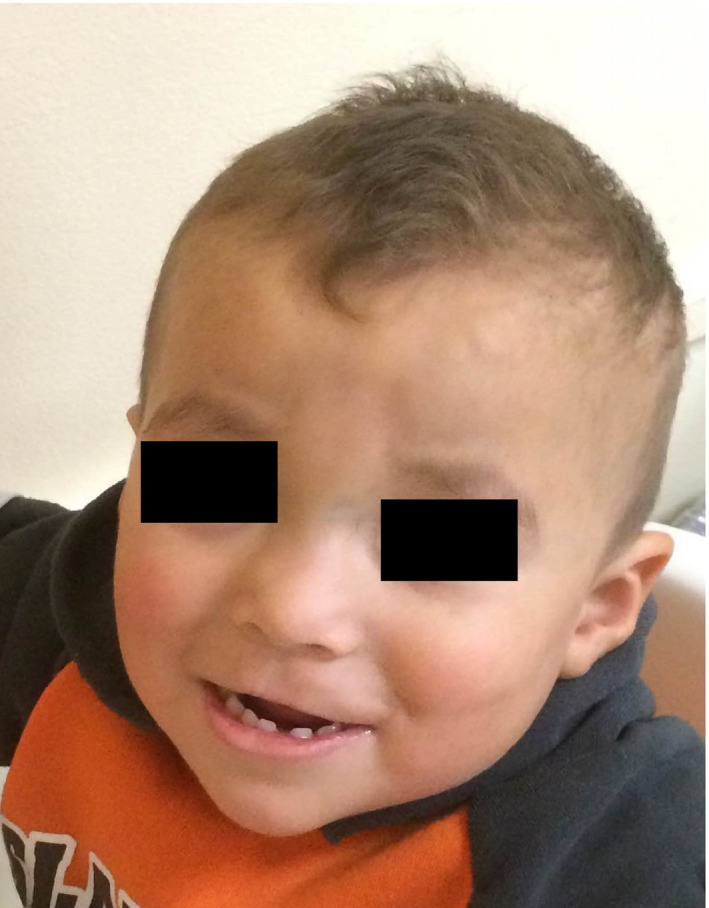
Low‐set ears, invasion of philtral skin onto the vermillion of the upper lip, and decreased scalp hair density.

On clinical examination, he was noted to have Blaschkoid hypo‐ and hyperpigmented streaks on his trunk as well as the upper and lower extremities (Fig. 1), a high forehead, wide‐set eyes, broad nasal root, low‐set ears, invasion of philtral skin onto the vermillion of the upper lip, and decreased scalp hair density (Fig. 2). Although the appearance of Blaschkoid pigmentary changes accompanied by neurologic issues was concerning for hypomelanosis of Ito, the overall constellation of findings was concerning for PKS. Dermatology performed two skin biopsies for fibroblast chromosome analysis, one from an affected (pigmented) area and a second from clinically normal skin. Of the 20 G‐banded cells/colonies examined, 55% contained an isochromosome 12p, consistent with the diagnosis of PKS.

## Discussion

Pallister–Killian syndrome is a rare, sporadic, multisystem disorder with distinct features, including facial abnormalities and organ system involvement. Although the prevalence is unknown, there have been more than 100 cases reported in the medical literature and the incidence of PKS increases with increasing maternal age 3, 4. Facial features in PKS are characteristic and very useful for diagnosis. They include fronto‐parietal alopecia, sparse eyebrows, long philtrum or invasion of the upper lip vermillion by philtral skin (the “Pallister lip” present in 100% of probands of PKS), depressed nasal bridge, large mandible, bifid uvula, and a short neck 5.

Pallister–Killian syndrome can involve any organ system in the body. Although there are no specific malformations that are pathognomonic of PKS, the clinical aberrations encountered are highly distinct. Additional characteristic features (Table 1) include the following: (1) neurologic manifestations such as structural brain malformations and epilepsy; (2) structural cardiac defects which may include atrial and ventricular septal defects; (3) pulmonary involvement, most commonly lung hypoplasia secondary to a diaphragmatic hernia; (4) gastrointestinal manifestations that may include intestinal malrotation, displacement of the anus; and (5) musculoskeletal abnormalities including a growth pattern unique to PKS which consists of an accelerated prenatal growth period followed by a decelerated postnatal growth period; seventy‐five percent of patients with PKS have some degree of visual impairment 3, 5. Congenital diaphragmatic hernia is thought to be very specific of PKS and is frequently a cause of death during the early postnatal period of life 6.

**Table 1:** Clinical Manifestations of PKS

System	Clinical manifestations
Neurologic	Structural brain malformations (60–70%)
	Epilepsy (53%) – myoclonic (56%), generalized convulsions (48%), clustered tonic spasms (30%)
Cardiac	Structural heart defects (40%)
	Atrial & ventricular septal defects (15%)
	Bicuspid aortic valve, aortic dilatation, patent ductus arteriosus, patent foramen ovale
Pulmonary	Diaphragmatic hernias with subsequent lung hypoplasia
Gastrointestinal (GI)	GI involvement (52%)
	Intestinal malrotation (12%)
	Congenital diaphragmatic hernia (11%)
	Umbilical hernias
	Displacement of anus
	Feeding difficulty
	Dysphagia
	Gastroesophageal reflux disease
	Constipation
Genitourinary	Cryptorchidism
	Hypospadias
	Small genitalia
	Hydrocele
Musculoskeletal	Polydactyly
	Joint contractures
	Hip dislocations
	Limb length discrepancies
Growth Pattern	Prenatal overgrowth – weight, length, and head circumference above 50th percentile
	Postnatal growth deceleration
Audiologic	Hearing loss (77%) – typically bilateral, sensorineural (38%), conductive (29%), and mixed (33%)
Ophthalmologic	Ocular involvement (87%)
	Visual impairment – strabismus, nystagmus, or myopia (75%)
	Legally blind (19%)
Dermatologic	Hypo‐/hyperpigmentation (45%)

Cutaneous findings are present in 45% of probands of PKS 5. These findings manifest as streaks or patches of hyperpigmentation or hypopigmentation and can appear as a whorled pattern following the lines of Blaschko. These cutaneous lesions can occur anywhere on the body and may be visible under Wood's lamp 7. They may be apparent at birth or they can present later in life. These cutaneous lesions can also be seen in hypomelanosis of Ito, a common differential diagnosis for PKS. Hypomelanosis of Ito is a neurocutaneous syndrome secondary to chromosomal mosaicism with cutaneous manifestations of hypopigmented whorls that also follow the lines of Blaschko. It is similarly associated with mental retardation, seizures, developmental delay, cleft palate, skeletal abnormalities, and diffuse alopecia, and differentiation of these two entities is dependent on cytogenetics. A diagnosis of hypomelanosis of Ito was ultimately excluded based on cytogenetics, which confirmed a diagnosis of PKS in our patient. In all patients presenting with cutaneous findings characteristic of whorled pigmentary changes, it is warranted to obtain a skin biopsy with subsequent karyotyping in order to detect mosaicisms as this seems to be a nonspecific clinical indicator of chromosomal mosaicisms, especially if a congenital anomaly syndrome or developmental delay is also present 8.

This genetic syndrome is a tissue‐limited mosaicism caused by tetrasomy 12p which is due to a small extra chromosome, an isochromosome, made up of two copies of the short (p) arm of chromosome 12 found in various cells of the body. These abnormal cells have four copies of the short arm of chromosome 12 instead of the two that are normally present. The effects of mosaic tetrasomy 12p are due to the genes carried on the extra chromosome that ultimately culminate in craniofacial, cardiovascular, renal, and additional systemic abnormalities 9.

Diagnosis can occur early in the prenatal period by chorionic villous sampling, amniocentesis, and cordocentesis 10. Early detection allows families to elect to terminate or continue the pregnancy as desired. PKS is commonly detected in skin fibroblasts and can often be missed due to the tissue‐specific nature of the isochromosome. It is typically not detected in rapidly dividing cells such as the peripheral blood cells, and there are limited reports of cases being identified in peripheral lymphocytes 7, 11, 12. The diagnosis is highest among amniocytes and bone marrow cells with a detection rate of 100%, followed by a detection rate of 50–100% in fibroblasts and 0–2% in lymphocytes 13. Fluorescent in situ hybridization (FISH) can be employed with chromosome 12‐specific DNA probes in order to identify isochromosome 12p in fibroblasts 14. An alternative method for a rapid, effective, and noninvasive diagnosis of this mosaicism can be achieved with a buccal smear, as there is a high detection rate of more than 50% in this tissue 15.

Many afflicted individuals die in utero or during the postnatal period, but a few may survive into their early twenties 9. PKS has an overall poor neurologic prognosis with significant mental and motor retardation which initially presents itself in infancy. Patients who survive past early life frequently may become bedridden, likely never speak, and can develop incontinence 9.

## Conclusion

Pallister–Killian syndrome is a sporadic, multisystem genetic disorder with hypo‐ and hyperpigmentation of the skin reflecting the mosaic chromosomal abnormality. Timely diagnosis of PKS is crucial as it poses a substantial emotional and financial strain on families. Patients with skin pigmentation changes along with other distinctive facial features should undergo a biopsy with subsequent karyotyping. In PKS specifically, fibroblast cells should undergo karyotyping as the isochromosome 12p occurs with low frequency in peripheral blood lymphocytes. Dermatologists will likely be consulted to assist with the diagnostic biopsy and should be able to recognize its features to aid with diagnosis and care of these patients.

## Authorship

RT and LS: drafted the manuscript. CH, MB, RT, and LS: edited, reviewed, and approved the final version of the manuscript.

## Conflict of Interest

The authors have no financial or other conflict of interests to disclose.

## References

[1] Pallister, P. D. , L. F. Meisner , B. R. Elejalde , et al. 1977. The Pallister mosaic syndrome [abstract]. Birth Defects Orig. Artic. Ser. 13(3B):103–110. 890087

[2] Teschler‐Nicola, M. , and W. Killian . 1981. Case report 72: mental retardation, unusual facial appearance, abnormal hair. Synd. Ident. 7:6–7.

[3] Izumi, K. , and I. D. Krantz . 2014. Pallister‐Killian syndrome. Am. J. Med. Genet. C Semin. Med. Genet. 166C:406–413. 25425112 10.1002/ajmg.c.31423

[4] Wenger, S. L. , M. W. Steele , and W. D. Yu . 1988. Risk effect of maternal age in Pallister (12p) syndrome. Clin. Genet. 34:181–184. 3180504 10.1111/j.1399-0004.1988.tb02860.x

[5] Wilkens, A. , H. Liu , K. Park , L. B. Campbell , M. Jackson , A. Kostanecka , et al. 2012. Novel clinical manifestations in Pallister‐Killian syndrome: comprehensive evaluation of 59 affected individuals and review of previously reported cases. Am. J. Med. Genet. A 158A:3002–3017. 23169767 10.1002/ajmg.a.35722

[6] Bergoffen, J. A. , H. Punnett , T. J. Campbell , A. J. III Ross , E. Ruchelli , and E. H. Zackai . 1993. Diaphragmatic hernia in tetrasomy 12p mosaicism. J. Pediatr. 122:603–606. 8463911 10.1016/s0022-3476(05)83546-2

[7] Choo, S. , S. H. Teo , F. M. Tan , M. H. Yong , and L. Y. Ho . 2002. Tissue‐limited mosaicism in Pallister‐killian syndrome ¾ a case in point. J. Perinatol. 22:420–423. 12082482 10.1038/sj.jp.7210712

[8] Ritter, C. L. , M. W. Steele , S. L. Wenger , and B. A. Cohen . 1990. Chromosome mosaicism in hypomelanosis of Ito. Am. J. Med. Genet. 35:14–17. 2301465 10.1002/ajmg.1320350104

[9] Schinzel, A. 1991. Tetrasomy 12p (Pallister‐Killian syndrome). J. Med. Genet. 28:122–125. 2002482 10.1136/jmg.28.2.122 PMC1016781

[10] Srinivasan, A. , and D. Wright . 2014. Pallister‐Killian syndrome. Am. J. Case Rep. 15:194–198. 24826207 10.12659/AJCR.890614 PMC4018245

[11] Horn, D. , F. Majewski , B. Hildebrant , and H. Korner . 1995. Pallister‐Killian syndrome: normal karyotype in prenatal chorionic villi, in postnatal lymphocytes, and in slowly growing epidermal cells, but mosaic trisomy 12p in skin fibroblasts. J. Med. Genet. 32:69–71. 10.1136/jmg.32.1.68 PMC1050184 7897632

[12] Conlin, L. K. , M. Kaur , K. Izumi , et al. 2012. Utility of SNP arrays in detecting, quantifying, and determining meiotic origin of tetrasomy 12p in blood from individuals with Pallister‐Killian syndrome. Am. J. Med. Genet. A 158A:3046–3053. 23169773 10.1002/ajmg.a.35726

[13] Jamuar, S. , A. Lai , S. Unger , and G. Nishimura . 2012. Clinical and radiological findings in Pallister‐Killian syndrome. Eur. J. Med. Genet. 55:167–172. 22387057 10.1016/j.ejmg.2012.01.019

[14] Speleman, F. , J. G. Leroy , N. Van Roy , et al. 1991. Pallister‐Killian syndrome: characterization of the isochromosome 12p by fluorescent in situ hybridization. Am. J. Med. Genet. 41:381–387. 1789295 10.1002/ajmg.1320410321

[15] Ohashi, H. , S. Ishikiriyama , and Y. Fukushima . 1993. New diagnostic method for Pallister‐Killian syndrome: detection of i(12p) in interphase nuclei of buccal mucosa by fluorescence in situ hybridization. Am. J. Med. Genet. 45:123–128. 8418650 10.1002/ajmg.1320450136

